# Neuromodulation via the Cerebrospinal Fluid: Insights from Recent *in Vitro* Studies

**DOI:** 10.3389/fncir.2018.00005

**Published:** 2018-02-05

**Authors:** Andreas Bjorefeldt, Sebastian Illes, Henrik Zetterberg, Eric Hanse

**Affiliations:** ^1^Department of Physiology, Institute of Neuroscience and Physiology, University of Gothenburg, Gothenburg, Sweden; ^2^Department of Neuroscience, Brown University, Providence, RI, United States; ^3^Department of Psychiatry and Neurochemistry, Institute of Neuroscience and Physiology, University of Gothenburg, Gothenburg, Sweden; ^4^Clinical Neurochemistry Laboratory, Sahlgrenska University Hospital, Gothenburg, Sweden; ^5^Department of Molecular Neuroscience, UCL Institute of Neurology, University College London, London, United Kingdom; ^6^United Kingdom Dementia Research Institute, University College London, London, United Kingdom

**Keywords:** cerebrospinal fluid, neuromodulation, neural circuit

## Abstract

The cerebrospinal fluid (CSF) occupies the brain’s ventricles and subarachnoid space and, together with the interstitial fluid (ISF), forms a continuous fluidic network that bathes all cells of the central nervous system (CNS). As such, the CSF is well positioned to actively distribute neuromodulators to neural circuits *in vivo* via volume transmission. Recent *in vitro* experimental work in brain slices and neuronal cultures has shown that human CSF indeed contains neuromodulators that strongly influence neuronal activity. Here we briefly summarize these new findings and discuss their potential relevance to neural circuits in health and disease.

## Introduction

**FIGURE 1:**
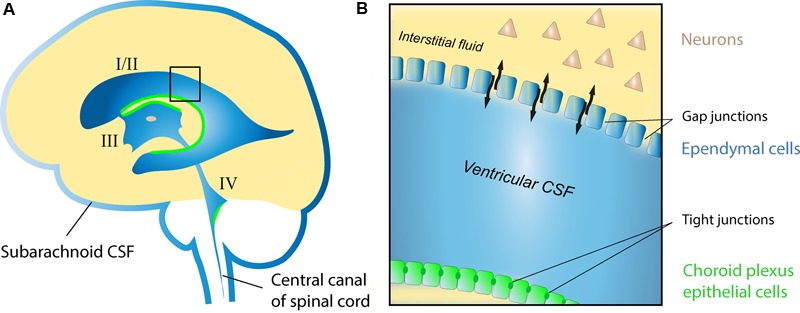
Schematic drawing of the human CSF system. **(A)** The position of the lateral (I/II), third (III), and fourth (IV) ventricles with respective choroid plexa (green), and the subarachnoid CSF compartments surrounding the brain and spinal cord. **(B)** Enlargement of area highlighted in **(A)** showing the interface between ventricular CSF and the ISF of the parenchyma in the adult brain. Note the restricted molecular exchange across the choroid plexus epithelial lining, where cells are adjoined by tight junctions (blood-CSF barrier), as opposed to areas of the ventricular lining composed of ependymal cells connected by gap junctions (black arrows indicate free exchange of molecules between CSF and ISF at these sites).

The cerebrospinal fluid (CSF) system is an evolutionarily preserved feature of animal brains (Brocklehurst, 1979) and provides central neurons with a regulated chemical environment well suited to promote their function and survival. CSF is a clear transparent extracellular fluid occupying the ventricles (I–IV) and subarachnoid space (**Figure 1A**), and forms a continuous fluidic network together with the interstitial fluid (ISF) of the parenchyma (**Figure 1B**). Its composition is characterized by low protein (∼1% of blood) and high salt (>150 mmol/L) content, and is influenced by multiple sources including blood, the choroid plexus, ventricular ependymal cells, neurons and glia (Smith et al., 2004; Skipor and Thiery, 2008).

Communicating freely with the ISF (Brightman and Palay, 1963; Smith et al., 2004), the CSF system has the potential to serve as a vessel for neuromodulatory signals acting via volume transmission (Agnati et al., 1986, 2010). However, whether the CSF system in fact plays an active, rather than simply passive, role in distributing neuromodulators throughout the brain is still unknown. In this review, we summarize a set of new experimental findings *in vitro* showing that endogenous neuromodulators in human CSF potently influence the function of pyramidal cells and interneurons in the rat hippocampal brain slice, and in rat cortical neuronal cultures. The potential significance of this neuromodulation is discussed in health and disease, and an outlook on the future advancement of this research field is provided.

## Recent *in Vitro* Findings with Human Cerebrospinal Fluid

### Electrophysiological Recordings from Rat Hippocampal Brain Slices

Since its introduction in the early 1970s, the hippocampal brain slice preparation has remained a major *in vitro* platform to study synaptic, cellular and network aspects of neuronal activity (Skrede and Westgaard, 1971). However, a limitation of this experimental model is that neurons in brain slices are perfused with artificial extracellular fluid (artificial CSF, aCSF) during recordings. Consisting simply of electrolytes, glucose and water, aCSF lacks the complex organic make-up of physiological CSF including proteins, peptides, lipids, amino acids, etc. Of particular relevance with respect to neuronal activity and function, physiological CSF is also known to contain a wide range of neuromodulators (**Table 1**) whose collective influence on cellular and synaptic properties has been poorly investigated.

**Table 1:** Examples of neuromodulators present in CSF and their respective targets in the central nervous system.

Neuromodulator	Target receptor(s)	Reference
**Classical transmitters**		
Acetylcholine	Nicotinic AChRs (ionotropic), M1–M5 muscarinic AChRs (GPCRs)	Welch et al., 1976
Dopamine	D1, D2, D3, D4, D5 (GPCRs)	Strittmatter et al., 1997
Histamine	H1, H2, H3 (GPCRs)	Gabelle et al., 2017
Noradrenaline	α1, α2, β1, β2 (GPCRs)	Strittmatter et al., 1997
Serotonin	5-HT3 (ionotropic), 5-HT1, 5-HT2, 5-HT4 – 5-HT7 (GPCRs)	Strittmatter et al., 1997
**Neuropeptides**		
Cholecystokinin	CCKA, CCKB (GPCRs)	Geracioti et al., 1993
Neuropeptide Y	Y1, Y2, Y4, Y5 (GPCRs)	Nilsson et al., 2001
Orexin	OX1, OX2 (GPCRs)	Heywood et al., 2015
Oxytocin	OXTR (GPCR)	Martin et al., 2014
Somatostatin	SSTR1, SSTR2, SSTR3, SSTR4, SSTR5 (GPCRs)	Nilsson et al., 2001
Substance P	NK1R (GPCR)	Juul et al., 1995
Vasoactive intestinal peptide	VPAC1, VPAC2 (GPCRs)	Juul et al., 1995
Vasopressin	V1a, V1b (GPCRs)	Martin et al., 2014
**Neurosteroids**		
Allopregnanolone	GABA_A_Rs, NMDARs (ionotropic)	Meletti et al., 2017
DHEAS	GABA_A_Rs, NMDARs (ionotropic)	Kim et al., 2003
Pregnenolone sulfate	GABA_A_Rs, NMDARs (ionotropic)	Kim et al., 2003
**Purines**		
Adenosine	(P1) A_1_, A_2A_, A_2B_, A_3_ (GPCRs)	Schmidt et al., 2015
Adenosine triphosphate	P2X_1_–P2X_7_ (ionotropic), P2Y_2_, P2Y_11_ (GPCRs)	Schmidt et al., 2015
**Endocannabinoids**		
2-AG	CB_1_, CB_2_ (GPCRs)	Romigi et al., 2010
Anandamide	CB_1_, CB_2_	Romigi et al., 2010
**Amino acids**		
GABA	GABA_A_Rs (ionotropic), GABA_B_Rs (GPCRs)	Orhan et al., 2017
Glutamate	NMDARs, AMPARs (ionotropic), mGluRs (GPCRs)	Spreux-Varoquaux et al., 2002
**Gasses**		
Nitric oxide	Guanylyl cyclase	Denda et al., 2011

*AChR, acetylcholine receptor; GPCR, G-protein coupled receptor; D1–5, dopamine receptors 1–5; H1–3, histamine receptors 1–3; α/β 1–2, alpha and beta noradrenergic receptors 1 and 2; 5-HT1–7, 5-hydroxytryptamine receptors 1–7; CCKA/B, cholecystokinin receptors A and B; Y1, Y2, Y4, Y5, neuropeptide y receptors 1, 2, 4, and 5; OX1–2, orexin receptors 1 and 2; OXTR, oxytocin receptor; SSTR1–5, somatostatin receptors 1–5; NK1R; neurokinin 1 receptor; VPAC1–2, vasoactive intestinal peptide receptors 1 and 2; V1a/b, vasopressin receptors V1a and V1b; GABA_*A/B*_Rs, types A and B gamma-aminobutyric acid receptors; NMDARs, *N*-methyl-D-aspartic acid receptors; A_*1*_, A_*2A*_/_*B*_, A_*3*_, adenosine receptors A_*1*_, A_*2A*_, A_*2B*_ and A_*3*_; P2X_*1*-*7*_/P2Y_*2/11*_; adenosine triphosphate (ATP) receptors P2X_*1*-*7*_, P2Y_*2*_ and P2Y_*11*_; CB_*1/2*_, cannabinoid receptors type 1 and 2; AMPARs, α-amino-3-hydroxy-5-methyl-4-isoxazolepropionic acid receptors; mGluRs, metabotropic glutamate receptors.*

**FIGURE 2:**
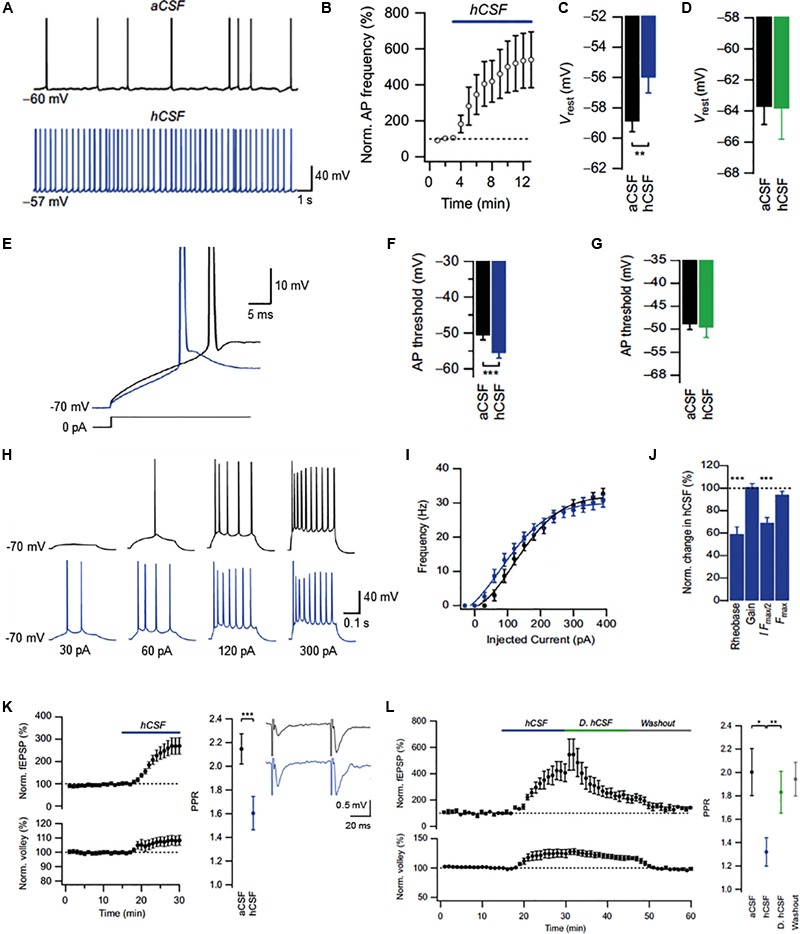
Effects of human CSF on CA1 pyramidal cells in the rat hippocampus. **(A)** Example traces of spontaneous action potentials recorded at resting membrane potential (Vrest) in aCSF and after 10 min of hCSF perfusion. **(B)** Summary graph of the effect of hCSF on spontaneous firing at Vrest. **(C,D)** Summary bar graphs showing effect of hCSF on Vrest under conditions of unperturbed **(C)** and clamped **(D)** G-protein activity. **(E)** Example traces of action potential threshold recorded in aCSF and after 10 min of hCSF perfusion. **(F,G)** Summary bar graphs showing effect of hCSF on action potential threshold under conditions of unperturbed **(F)** and clamped **(G)** G-protein activity. **(H)** Example traces of action potentials evoked by depolarizing current injections from –70 mV in aCSF and after 10 min of hCSF perfusion. **(I)** Summary graph of the effect of hCSF on the frequency-current (input–output) relationship in CA1 pyramidal cells. **(J)** Summary graph showing normalized change in rheobase, gain (slope), amount of injected current required to reach 50% of maximum firing frequency (I *F*_max/2_) and maximum firing frequency (*F*_max_) in hCSF. **(K)** Summary graph showing effect of hCSF on the EPSP slope (upper left graph), fiber volley (lower left graph), and paired-pulse ratio (PPR) in extracellular field recordings from CA1 stratum radiatum. **(L)** Summary graph of the effect of normal vs. dialyzed hCSF (hCSF and D. hCSF) on the EPSP slope (upper graph) and fiber volley (lower graph) in extracellular field recordings. ^∗^*P* < 0.05; ^∗∗^*P* < 0.01; ^∗∗∗^*P* < 0.001. Adapted from Bjorefeldt et al. (2015).

In a recent study, Bjorefeldt et al. (2015) used the simplistic make-up of aCSF to establish the functional impact of endogenous neuromodulators in real brain extracellular fluid, i.e., human CSF (hCSF), on rat hippocampal neurons. The study measured electrolyte and glucose levels in pooled samples of hCSF obtained by lumbar puncture from both neurological patients and healthy volunteers. A matched aCSF was then designed based on obtained measurements and used as control for potential neuromodulatory effects of hCSF on hippocampal neurons. In whole-cell patch clamp recordings from CA1 pyramidal cells, hCSF caused a strong increase in neuronal excitability, boosting spontaneous action potential (AP) firing approximately fivefold (**Figures 2A,B**). Moreover, hCSF depolarized the resting membrane potential of CA1 pyramidal cells (**Figures 2A,C**) and lowered their firing threshold (**Figures 2E,F**) through apparent G-protein signaling-dependent mechanisms (**Figures 2D,G**), leading to a left shifted frequency-current (input–output) relationship (**Figures 2H–J**). In extracellular field recordings from CA3–CA1 synapses in stratum radiatum, hCSF caused a large increase in evoked excitatory synaptic transmission that was accompanied by an apparent increase in presynaptic release probability (**Figure 2K**). In attempt to isolate the active neuromodulatory fraction of hCSF based on molecular size, the authors dialyzed hCSF samples to remove all constituents larger than 8 kDa. Compared to untreated hCSF, evoked excitatory synaptic responses were strongly reduced in the dialyzed hCSF, indicating a large contribution from low molecular weight (≤8 kDa) substances to these effects (**Figure 2L**). Overall the study revealed strong neuromodulatory influence of hCSF on hippocampal CA1 pyramidal cells, suggesting that such neuromodulation could be of relevance to pyramidal cell function *in vivo*.

**FIGURE 3:**
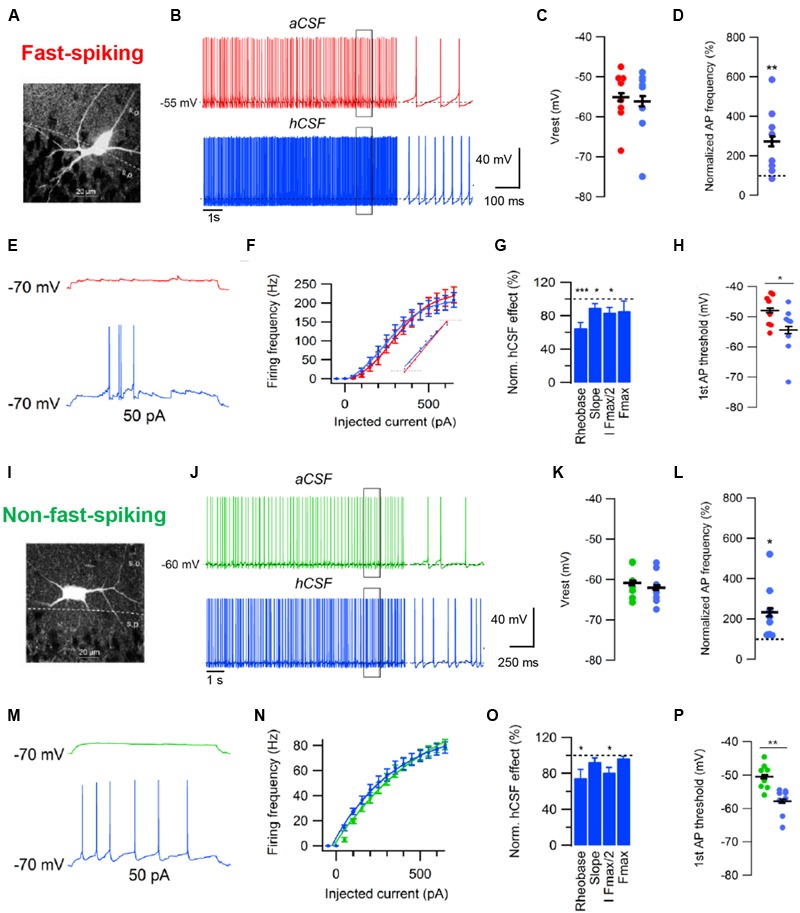
Effects of human CSF on fast-spiking and non-fast-spiking rat hippocampal CA1 interneurons. **(A)** Example of cell morphology in the FS interneuron group. **(B)** Example traces of spontaneous action potentials recorded at Vrest in aCSF and after 10 min of hCSF perfusion in a FS interneuron. **(C,D)** Summary bar graphs showing effect of hCSF on Vrest **(C)** and spontaneous firing frequency **(D)** in the FS interneuron group. **(E)** Example traces of action potentials evoked by depolarizing current injection from –70 mV in aCSF and after 10 min of hCSF perfusion. **(F)** Summary graph of the effect of hCSF on the frequency-current (input–output) relationship in FS CA1 interneurons. **(G)** Summary graph showing normalized change in rheobase, gain (slope), amount of injected current required to reach 50% of maximum firing frequency (I *F*_max/2_) and maximum firing frequency (*F*_max_) in hCSF. **(H)** Summary graph of the effect of hCSF on the threshold of first elicited action potential in response to depolarizing current injection from –70 mV in FS interneurons. **(I)** Example of cell morphology in the NFS interneuron group. **(J)** Example traces of spontaneous action potentials recorded at Vrest in aCSF and after 10 min of hCSF perfusion in a NFS interneuron. **(K,L)** Summary bar graphs showing effect of hCSF on Vrest **(K)** and spontaneous firing frequency **(L)** in the NFS interneuron group. **(M)** Example traces of action potentials evoked by depolarizing current injection from –70 mV in aCSF and after 10 min of hCSF perfusion. **(N)** Summary graph of the effect of hCSF on the frequency-current (input–output) relationship in NFS CA1 interneurons. **(O)** Summary graph showing normalized change in rheobase, gain (slope), amount of injected current required to reach 50% of maximum firing frequency (I *F*_max/2_) and maximum firing frequency (*F*_max_) in hCSF. **(P)** Summary graph of the effect of hCSF on the threshold of first elicited action potential in response to depolarizing current injection from –70 mV in NFS interneurons. ^∗^*P* < 0.05; ^∗∗^*P* < 0.01; ^∗∗∗^*P* < 0.001. Adapted from Bjorefeldt et al. (2016).

Another recent study examined the effects of hCSF on GABAergic interneurons in CA1 stratum pyramidale of rat (Bjorefeldt et al., 2016). Cortical GABAergic interneurons are a diverse population consisting of many subtypes of cells with distinct anatomical and physiological properties (Klausberger and Somogyi, 2008; Moore et al., 2010; Tremblay et al., 2016). Bjorefeldt et al., 2016 examined two broad groups interneurons that were classified as having either fast-spiking (FS) or non-fast-spiking (NFS) phenotype based on their voltage response to a series of depolarizing and hyperpolarizing current injections (Bjorefeldt et al., 2016). The authors found that hCSF, again compared to a matched aCSF, increased the excitability of both groups of CA1 interneurons, boosting their spontaneous firing two–threefold over 10 min in whole-cell current clamp recordings (**Figures 3A,B,D,I,J,L**). However, in contrast to CA1 pyramidal cells, hCSF had no effect on the resting membrane potential of interneurons (**Figures 3B,C,J,K**), indicating cell specificity of the neuromodulatory effects. Similar to pyramidal cells, hCSF caused a left-shift in the frequency-current relationship of both groups of interneurons, increasing their responsiveness to excitatory input (**Figures 3E–H,M–P**). Moreover, both interneuron groups displayed increased firing in response to sinusoidal current injections at theta and gamma frequencies in hCSF. Together with strong excitation of pyramidal cells, these effects are likely to promote hippocampal oscillatory network activity (Bjorefeldt et al., unpublished findings).

Together these studies show that (i) hCSF contains physiologically active neuromodulators that potently increase the excitability of both pyramidal cells and interneurons *in vitro*, (ii) some of these neuromodulatory effects are cell-type specific and act through G-protein coupled receptors (GPCRs), (iii) pyramidal cells appear to be more strongly modulated than the two examined groups of GABAergic interneurons, (iv) active neuromodulators are largely ≤ 8 kDa in size and (v) the presence of neuromodulators in physiological brain extracellular fluid such as hCSF can help explain differences in the amount of spontaneous neuronal activity observed *in vivo* vs. in typical *in vitro* brain slice recordings.

### Multielectrode Array Recordings from Rodent Primary and Stem Cell-Derived Neural Cultures

**FIGURE 4:**
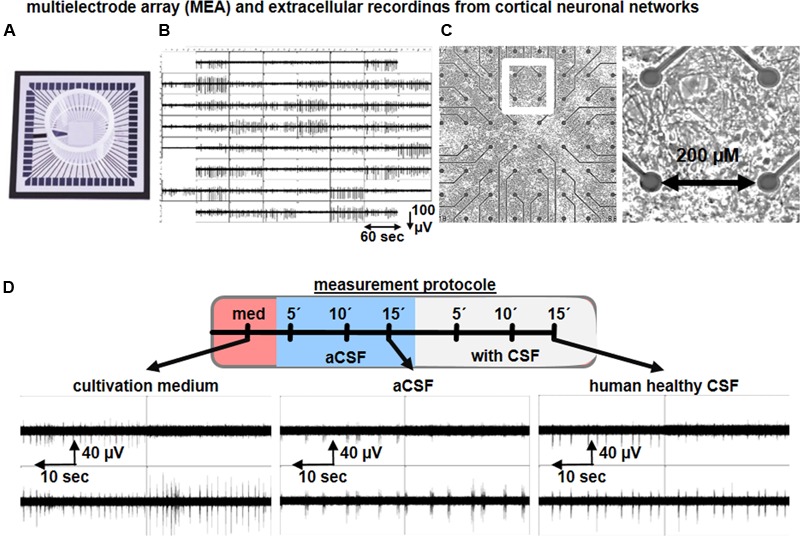
Microelectrode array recordings of neuronal culture to study the impact of human CSF on neuronal network activity. Representative image of a **(A)** multielectrode array (MEA), **(B)** synchronous neuronal network activity detected by 59 electrodes, **(C)** cortical neural cells cultures on MEAs, and cortical neuronal morphology. **(D)** Schematic drawing of a recording procedure used to study the functional impact of human CSF samples on neuronal cultures. Examples show the neuronal network activity of cortical neurons exposed to cultivation media, aCSF and human CSF samples from a healthy individual.

Multielectrode arrays (MEAs) are extracellular recording devices allowing the detection of neuronal network activity generated by cultured neurons or within brain slice preparations (**Figure 4**). MEAs are composed of tens of electrodes embedded into a glass or plastic substrate. On this electrode array, neural cells can be cultured and brain slice preparations can be placed to study neuronal activity at the single cell and network level for minutes up to several months (Obien et al., 2014). For this purpose, MEAs are connected to an amplifier and computer system, where an increasing amount of data analysis approaches is being used to analyze the electrophysiological neuronal activity. The extracted neuronal signals are comprised of spikes (filtered signals > 200 Hz) and local field potentials (filtered signals < 200 Hz), and thereby, neuronal function can be analyzed at different levels in a non-invasive approach. As the extracellular solution in the recording chamber is easily exchangeable, MEAs represent an ideal non-invasive measurement tool to understand the acute and chronic impact of CSF samples on single neurons as well as neuronal networks.

Otto et al. (2009) described the neuronal network function of rodent primary tissue-derived, and stem cell-derived, neurons exposed to aCSF or to hCSF samples obtained from healthy individuals and traumatic brain injury (TBI) patients by applying MEAs. The mouse embryonic stem cell (mESC)-derived neuronal population used comprises ∼70% GABAergic neurons (Illes et al., 2009) and ∼70% of neurons are intrinsically active (Illes et al., 2014). Due to this cellular composition, the mESC-derived neuronal cultures are predominantly asynchronously or partially synchronously active in aCSF or cultivation media. However, substitution of aCSF with healthy hCSF produces highly synchronous neuronal network activity. As described by Otto et al. (2009), several network activity parameters are increased after healthy hCSF application, such as the number of spikes, population bursts and parameters to describe synchrony such as Cohen’s kappa value. Since hippocampal and cortical rodent neuronal cultures are predominantly of glutamatergic identity, the recorded network activity in the presence of culture media or aCSF is highly synchronous (Wagenaar et al., 2006; Illes et al., 2014; Perez-Alcazar et al., 2016) (**Figure 4D**). Nevertheless, hippocampal or cortical neuronal cultures exposed to healthy hCSF show a strong increase in the number of population bursts (Otto et al., 2009; Gortz et al., 2013) (**Figure 4D**). Thus, all together these studies show that hCSF enhances neuronal activity and induces synchronicity in the network (Otto et al., 2009; Gortz et al., 2013; Jantzen et al., 2013), which is consistent with the results obtained in hippocampal brain slices.

### Study Limitations

There are a number of outstanding questions and limitations associated with above described studies. Perhaps most notable is that the specific identity of active neuromodulators in hCSF, as well as their respective receptor signaling pathways, remains to be identified. A further limitation is that effects of hCSF were examined in neurons from rat. Thus, to what extent are findings with hCSF in these studies of relevance to the human brain? To control for potential species differences in neuromodulatory effects we attempted to sample CSF from the cisterna magna of rats. Unfortunately, given the small extractable volume from each rat (∼150 μL) we were unable to obtain a CSF pool large enough to allow proper electrophysiological experimentation. Since neuromodulatory systems architecture is known to be well conserved across species (O’Connell, 2013; Swallow et al., 2016; Lovett-Barron et al., 2017), we would expect hCSF to produce similar effects on human hippocampal and cortical neurons. In line with this prediction, a recent study using human neocortical brain slices prepared from resected tissue of epilepsy patients reported that hCSF, as compared to artificial culturing media, enhanced neuronal activity (Schwarz et al., 2017).

Another issue relates to the artificial manner in which neurons were exposed to neuromodulators in these studies. hCSF was introduced directly to brain slices and cultured neurons via an artificial perfusion system, allowing neuromodulators to bypass any natural diffusion barrier. It therefore remains unclear whether certain neuromodulators in hCSF would ever reach the corresponding areas in the intact brain. Moreover, it is possible that the neuromodulatory composition of hCSF could differ between brain and spinal compartments. All hCSF used in above described studies was sampled from the lumbar subarachnoid space, raising the question of whether hCSF sampled, e.g., from one of the ventricles could have produced somewhat different effects.

Finally, a number of studies suggest that the CSF system during development is more compartmentalized than in the adult and young adult brain due to the presence of strap junctions between ependymal cells that largely limit the exchange of molecules between the CSF and ISF (Saunders et al., 2000; Whish et al., 2015). Thus, it should be noted that the findings with adult hCSF in above described studies may not have significant implications for neuromodulatory CSF signaling during development.

## Properties and Potential Significance of CSF Neuromodulation

The essential finding that hCSF contains physiologically active neuromodulators at ambient levels can be interpreted in light of two separate, although not mutually exclusive, scenarios. One possibility is that the neuromodulators, whose specific identities and relative functional importance are yet to be fully established, were selectively secreted into the CSF system in order to exert effects at downstream target areas. In this case, one could consider the CSF as an ‘active’ vessel or distributor of neuromodulatory signals within the brain, as has been previously suggested (Skipor and Thiery, 2008; Veening and Barendregt, 2010; Fuxe et al., 2012). An alternative scenario, considering the free bidirectional exchange of molecules between the CSF and ISF, is that the CSF acts as a sink for neuromodulatory substances originating from the neuronal parenchyma, diffusing away from their site of release along their concentration gradients. The CSF could then be viewed as reflecting an overall neuromodulatory environment with broad spatiotemporal profile, perhaps merely transporting neuromodulators that are to be cleared from the brain parenchyma.

Whether neuromodulators are in fact actively secreted into the CSF *in vivo* or not, their remarkably potent effects on cortical neurons in recent studies have interesting implications for neuromodulation and neuronal communication in the brain. These findings show that (i) there exists neuromodulatory substances in brain extracellular fluid that retain physiological function over prolonged time periods (up to several hours in our studies) and (ii) there are neuromodulatory GPCRs (not excluding contributions from other receptor types) in cortex with sufficient affinity for these neuromodulators to mediate observed effects on neuronal activity. Thus, these results provide strong support for the concept of long distance (non-synaptic) volume transmission (Agnati et al., 1986, 2010) as a means for neuronal communication in the brain. In line with our findings, neuromodulators acting via CSF volume transmission *in vivo* would likely operate on a slow (minutes to hours) timescale with broad spatial distribution, targeting neuromodulatory GPCR families (Fuxe et al., 2012, 2013). In addition to canonical intracellular metabotropic signaling, GPCR activation may influence ionotropic receptor families via heterodimerization (Borroto-Escuela et al., 2010, 2017).

Given the wide range of neuromodulators found in CSF (see **Table 1**), their collective influence on neural circuits should be considered. In such a paradigm, the functional impact of a given neuromodulator would depend on its local concentration, target receptor affinity, expression and distribution, as well as sensitivity to enzymatic degradation and/or uptake by transporter proteins. Moreover, parameters affecting diffusion through extracellular space, such as local tissue tortuosity and chemical properties of the neuromodulator (e.g., molecular weight and charge distribution), are likely to be important (Sykova, 2004). An interesting aspect of multiple neuromodulators acting via volume transmission to exert simultaneous influence on neural circuits is the potential for synergistic neuromodulatory effects, e.g., through converging intracellular signaling pathways.

Our recent findings have indicated that a major functional effect of CSF-distributed neuromodulators on cortical neurons is to boost intrinsic excitability and increase neuronal responsiveness to excitatory input. It is possible that these effects, causing a left-shift in neuronal input–output function, are indicative of how neuromodulation directs neurons and circuits into an efficient ‘online’ information processing state during wakefulness. The neuromodulatory effects of hCSF could facilitate and/or support multiple aspects of neural circuit function *in vivo*. For example, allowing fewer numbers of simultaneously active synapses to drive AP output would support a sparse and energy efficient information coding regime, which is thought to be utilized by the brain (Olshausen and Field, 2004; Wolfe et al., 2010; Barth and Poulet, 2012; Palm, 2013). With respect to learning and memory, the increased excitability of cortical pyramidal cells is likely to enhance NMDA receptor-mediated coincidence detection and synaptic plasticity in neural circuits through membrane potential depolarization and increased numbers of back-propagating APs (Tsubokawa and Ross, 1997; Lisman and Spruston, 2005). This would promote learning-associated structural and functional modifications to neural circuits during active wake, which is when synapses are thought to undergo strengthening to support acquisition and storage of new information (Tononi and Cirelli, 2014). In cortical networks, periods of synchronized fast GABAergic inhibition onto principal cells give rise to rhythmic membrane potential fluctuations in neuronal populations (network oscillations) that are hypothesized to facilitate information processing and cognition (Singer, 1999; Buzsaki, 2006; Lisman and Jensen, 2013). By boosting the excitability of both GABAergic interneurons and principal cells, it is likely that CSF neuromodulation would promote such rhythm generation in cortical networks and circuits.

In summary, in terms of its functional effects on neurons, this type of neuromodulation seems to support multiple circuit mechanisms hypothesized to underlie cognitive abilities. As an ambient form of neuromodulation it may serve to promote and/or facilitate signature neuronal activity patterns important in information coding and processing by neural circuits. Neuromodulatory effects of other spatiotemporal characteristics would be able to operate on top of such an ambient component, further enriching the means by which neuromodulation can regulate neural circuit function.

## Origin and Identity of CSF Neuromodulators

The chemical composition of CSF is shaped by contributions from multiple sources including blood, the choroid plexus, ependymal cells, neurons and glia (Smith et al., 2004; Skipor and Thiery, 2008). With respect to neuromodulators, release from CNS resident cells is likely to be a major source but the relative contribution of different CNS compartments is largely unknown. Part of the neuromodulatory composition of CSF could result from the tonic activity of subcortical monoaminergic and cholinergic nuclei during wakefulness, resulting in widespread release of noradrenaline, histamine, dopamine and serotonin and acetylcholine throughout the brain (Saper et al., 2001). The finding that release often occurs from varicosities lacking a postsynaptic element (Descarries and Mechawar, 2000; Vizi et al., 2010), and that there is mismatch in transmitter release site vs. receptor location (Jansson et al., 2002; Fuxe et al., 2005), suggests that monoamines and acetylcholine operate as volume transmitters in the CNS. Depending on local regulation of reuptake mechanisms and acetylcholine esterase levels, these classical transmitters could be active over long distances. Another potential source is the CSF-contacting neurons found in periventricular brain regions of a wide range of species. Some of these neurons feature varicose neuromodulator-containing processes that terminate in ventricular CSF (Parent, 1981; Vigh et al., 2004; Veening and Barendregt, 2010), suggesting that neuromodulators may be actively released into the CSF to exert downstream effects on target neurons. Dendritic neuropeptide release from large dense-core vesicles, such as seen in hypothalamic neuronal populations (Ludwig and Leng, 2006), may additionally contribute to the neuromodulatory composition of CSF. In addition to CNS derived release, certain blood-borne neuromodulators such as leptin have been shown to enter the CSF by way of specific transporters expressed at the choroid plexus blood-CSF barrier (Zlokovic et al., 2000; Skipor and Thiery, 2008). Furthermore, choroid plexus epithelial cells are known to secrete certain neuropeptides and neurotrophic factors into the CSF (Chodobski and Szmydynger-Chodobska, 2001; Smith et al., 2004).

Our work so far has focused largely on characterizing the functional impact of CSF neuromodulators on cortical neurons. While we believe this to be an important effort, the specific identities of active neuromodulators in hCSF should also be thoroughly addressed. There is at present evidence suggesting critical contribution from a distinct receptor subfamily in promoting hippocampal network oscillations *in vitro* (Bjorefeldt et al., unpublished findings). Depending on the experimental readout of neuronal activity (synaptic, cellular, network), and the type of neuronal population or brain area examined, the relative importance of specific neuromodulators in CSF and their various receptor signaling pathways may vary.

It can be concluded that any neuromodulator remaining functionally active at the level observed in present studies is remarkably resistant to both *in vivo* degradation and reuptake, and experimental handling such as thawing and freezing. Neuropeptides may display long half-lives in brain extracellular fluid and are active at nanomolar concentrations due to their high affinity GPCRs (Jones and Robinson, 1982; Ludwig and Leng, 2006; van den Pol, 2012). The actions of peripherally secreted peptides such as leptin and ghrelin on central neurons further suggest plausibility of neuropeptide involvement in the neuromodulatory effects of hCSF. Interestingly, some neuropeptides have also been shown to enhance effects of non-peptide neuromodulators such as acetylcholine (Mancillas et al., 1986; Kouznetsova and Nistri, 2000). Whether such results exemplify synergistic or additive neuromodulatory effects, this provides a mechanism by which multiple neuromodulators present at low ambient concentrations could significantly influence the operation of neural circuits *in vivo*.

## CSF Neuromodulators in Disease

The most studied neuromodulators in CSF in relation to disease are the classical transmitters dopamine, serotonin, acetylcholine, histamine, and noradrenaline. A prototype neurodegenerative disease in which neuromodulation is impaired is Parkinson’s disease; neurodegeneration-mediated depletion of dopamine in the striatum (especially the putamen) is the defining feature of the disorder, which has translated into a clinically established therapy based on levodopa/carbidopa treatment (Carlsson, 2002). Given the dopaminergic lesion, measurements of CSF concentrations of dopamine or its metabolites should provide a relatively straightforward diagnostic test but so far this expectation has not been realized. Nevertheless, some reports have noted decreased homovanillic acid (HVA, the end-product of dopamine metabolism) concentrations in CSF from patients with Parkinson’s disease (Zubenko et al., 1986; Chia et al., 1993; Loeffler et al., 1995), or decreased CSF concentrations of dihydroxyphenylacetic acid (DOPAC; the main neuronal metabolite of dopamine) (Zubenko et al., 1986; Chia et al., 1993; Gonzalez-Quevedo et al., 1993; Eldrup et al., 1995). These results were not confirmed in the deprenyl and tocopherol antioxidative treatment of parkinsonism (DATATOP) trial that reported negative results for both CSF HVA and DOPAC in Parkinson’s disease (LeWitt et al., 1992).

There are many potential reasons for why dopamine metabolite concentrations in CSF are not robustly associated with Parkinson’s disease. CSF HVA is only distantly related to neuronal dopamine stores and reflects several intervening processes. Since dopaminergic neurons do not contain catechol-*O-*methyltransferase, CSF HVA depends on uptake and intracellular *O*-methylation in non-dopaminergic cells. Thus, in Parkinson’s disease, the striatal content of HVA is not as severely decreased as that of dopamine (Lloyd et al., 1975).

Cerebrospinal fluid dopamine concentration may also not be a direct marker of central dopamine deficiency; increased dopamine release from remaining nerve terminals may compensate the loss of dopaminergic neurons in Parkinson’s disease, thereby augmenting dopamine delivery from those terminals to the extracellular fluid (Sossi et al., 2002). CSF dopamine concentration may thus underestimate the extent of loss of neuronal dopamine stores.

Cerebrospinal fluid DOPAC may be a superior marker of central dopamine stores, compared to HVA or dopamine itself. DOPAC is formed from deamination of cytosolic dopamine catalyzed by monoamine oxidase-A. Dopamine leaks continuously from vesicular stores into the cytosol. Therefore, the rate of DOPAC formation should be related to the amount of stored dopamine. Post-mortem putamen from patients with end-stage Parkinson’s disease shows similarly decreased tissue concentrations of dopamine and DOPAC (Goldstein et al., 2011), and CSF DOPAC is related directly to brain tissue content of this metabolite (Palfreyman et al., 1982).

Although several reports have noted low CSF DOPAC in Parkinson’s disease, these were relatively small studies (Goldstein, 2013). Further, they did not compare CSF DOPAC levels in other neurodegenerative diseases and it is presently unclear whether CSF DOPAC reduction is specific to Parkinson’s disease or not. More research on these topics is needed.

Other diseases in which neuromodulators play a major role for the clinical presentation are narcolepsia (orexin), Alzheimer’s disease (acetylcholine), neuroinflammatory conditions, including multiple sclerosis (cytokines and interleukins), schizophrenia (dopamine and its metabolites) and affective disorders (serotonin and its metabolites). CSF orexin concentration is used as an adjunct in the criteria of the disease [CSF orexin concentration is low in the disease due to the loss of orexin-producing neurons (Bourgin et al., 2008)]. For Alzheimer’s disease and affective disorders, changes in the neuromodulator concentrations are not specific enough to be clinically meaningful.

## Toward a Better Understanding of the Role of the CSF System in Neuromodulation

Neuromodulation encompasses a vast variety of biochemical processes, occurring at various spatiotemporal scales, which tune the excitability and function of central neurons. In this review, we have considered how long distance neuromodulation via the CSF might influence the function of neurons and neural circuits in health and disease, in light of new experimental findings. Further efforts are needed in order to establish whether the CSF acts as an ‘active’ neuromodulatory channel *in vivo* and, if so, how this neuromodulation impacts neural circuit function, e.g., during different brain states such as sleep and wake. To experimentally confirm such active role of the CSF system would require *in vivo* demonstration of (i) active release of neuromodulator into a CSF compartment, (ii) neuromodulator utilization of the CSF/ISF system to distribute within the brain and (iii) a functional impact of the neuromodulator(s) on a distant neuronal population. Moreover, it would require the capacity to distinguish between functional effects caused by CSF-derived and locally released neuromodulator.

A conceivable path toward elucidating the role of the CSF system in neuromodulation involves further research into the brain’s CSF-contacting neurons. Identification of cell type-specific markers enabling selective manipulation of CSF-contacting neurons that harbor neuromodulatory release machinery would greatly aid in this effort. The development of better techniques for detecting neuromodulator release and diffusion *in vivo* combined with use of, e.g., fluorescent GPCR activation reporters and genetically encoded calcium/voltage indicators should provide new and better means to address the functional relevance of neuromodulation via the CSF system in health and disease. At present, further *in vitro* studies examining the neuromodulatory influence of healthy and pathological CSF on central neurons and circuits are warranted.

## Author Contributions

All authors took part in designing, writing and revising the manuscript. AB and SI provided the data and prepared the figures and tables.

## Conflict of Interest Statement

The authors declare that the research was conducted in the absence of any commercial or financial relationships that could be construed as a potential conflict of interest. The reviewer GK and handling Editor declared their shared affiliation.
